# Characterization of quality parameters and phytosterol content in oils and their formulated margarines

**DOI:** 10.1002/fsn3.4437

**Published:** 2024-10-29

**Authors:** Anh T. L. Nguyen, Aleksei Kaleda, John O. Onuh, Alberta N. A. Aryee

**Affiliations:** ^1^ Food Science and Biotechnology Program, Department of Human Ecology, College of Agriculture, Science and Technology Delaware State University Dover Delaware USA; ^2^ Center of Food and Fermentation Technologies (TFTAK) Tallinn Estonia; ^3^ Department of Food and Nutritional Sciences Tuskegee University Tuskegee Alabama USA

**Keywords:** edible oils, fatty acid composition, margarine, oxidative stability, phytosterols

## Abstract

Unlike lipid stability and oxidation studies in commonly used edible oils and margarines, margarines formulated with unconventional oils are not well characterized. This study investigated the effect of heat treatment (HT) on the stability and content of phytosterol in njangsa seed oil (NSO), bush mango oil (BMO), soybean oil (SBO), coconut oil (CCO), and margarines formulated from their blends: BN (BMO and NSO), BS (BMO and SBO), CN (CCO and NSO), CS (CCO and SBO), and commercial margarines (CM1 and CM2). Both oils and margarines were heat‐treated at 130, 170, and 210°C for 10, 15, 20, and 120 min (only oils). Changes in free fatty acid (FFA), peroxide value (PV), para‐anisidine value (AnV), and fatty acid (FA) composition and phytosterol content were determined after 20 min (margarines) and 120 min (oils) of HT and compared to their control/pre‐HT/unheated (UH) counterparts. The FA composition did not change significantly with HT. Polyunsaturated fatty acids (PUFA)‐rich oils such as NSO and SBO showed significantly higher increase in FFA content with HT compared with oils with higher saturated fatty acid content (BMO and CCO). Oils with higher proportions of linoleic acid, such as SBO (68.3%) and NSO (60.35%), had higher AnV at the end of the HT compared with oils with lower content, such as BMO (0.51%). Phytosterol content fluctuated with HT, and changes in content were generally more pronounced in β‐sitosterol than in stigmasterol and campesterol. Both principal component analysis (PCA) and partial least‐squares discriminant analysis (PLS‐DA) were carried out to observe possible clusters. The results suggest that changes in quality and content of oils and margarines during heating are dependent on more than their fatty acid composition.

## Graphical abstract

The quality parameters and phytosterol content of oils and margarines were modified with heat treatment and these changes were quantified using titrimetric and chromatographic methods.

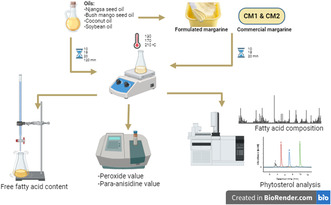

## 1 INTRODUCTION

Vegetable oils and margarines (water‐in‐oil emulsion) are important and versatile dietary staples. Changes in their properties and several undesirable processes, such as oxidation, hydrolysis, isomerization, and polymerization, may be accelerated when exposed to process conditions (e.g., frying, baking, and other applications) and in the presence of oxygen and moisture (Shin et al., 2021; Silva et al., 2022). These changes can affect their sensory, nutritional, and functional properties and may negatively impact health (Silva et al., 2022). The intensity of lipid oxidation is influenced by the type of oil/margarine, fatty acid composition, presence of anti‐ and prooxidants, manufacturing, process and storage conditions (Silva et al., 2021).

The adoption of strategies such as blending and interesterification to formulate *trans*‐free and polyunsaturated fatty acids (PUFA)‐rich margarines and other products are increasingly becoming popular due to current knowledge and regulation on *trans* and saturated fatty acids (SFA) (Aryee et al., 2022; Ke et al., 2024), using some common as well as novel/unconventional oils and fats (Wongjaikham et al., 2022; Yirankinyuki et al., 2018). Oils from njangsa (*Ricinodendron heudelotii*) seed, bush mango (*Irvingia gabonensis*) kernel, soybean, and coconut have unique fatty acid profiles and are rich sources of functional components such as polyphenolic compounds, antioxidants, and other health‐promoting compounds including phytosterols (Akonjuen et al., 2023; Arrey et al., 2022; Lykke et al., 2021; Ogunsina et al., 2012; Yeboah et al., 2011). Blending PUFA‐rich oils with high content of SFA or monounsaturated fatty acids (MUFA) established a probable solution to improve oil stability, specifically in high‐heat cooking oils (Ben Hammouda et al., 2018; Sivakanthan et al., 2023).

However, these naturally occurring components may be degraded and oxidized during thermal treatment, thereby reducing their quality and value (Ullah et al., 2020). The phytosterol content in rapeseed oil was increased in the first 2 h of heating and then decreased with extended heating time (Kasprzak et al., 2020), while the evolution of oxidized phenolic compounds was observed in heated olive oil (Daskalaki et al., 2009). However, in another study of rapeseed oil, the content of individual and total phytosterol steadily decreased during the 24–48 h heating process and varied depending on the form of used oil (pressed, refined, and partially hydrogenated IV = 90/70) (Kmiecik et al., 2020).

Unlike palm oil fractions which have been blended with other oils to produce margarine and are fairly studied, assessment of the stability of unconventional oils such as from njangsa seed and bush mango kernel and their blends is elusive. Due to the high PUFA content of some of these oils and the high content of fats in margarines (80%), they are prone to oxidation which affects their shelf‐life stability and usefulness. This study aims to determine the pattern of change in quality parameters by measuring the oxidative stability, fatty acid composition, and phytosterol content of njangsa, bush mango, soybean, and coconut oils and margarines formulated from their blends under controlled heat treatment. Principal component analysis (PCA), partial least‐squares discriminant analysis (PLS‐DA), and clustering were used to reduce the dimensionality of the dataset into a few layers of key features and identify patterns in the dataset by grouping observations.

## 2 MATERIALS AND METHODS

### 2.1 Materials

Njangsa seed and bush mango kernel were purchased from a local market in Mamfe, Cameroon. Organic coconut and soybean oils and two commercial margarines were purchased from a local supermarket in Dover, DE. The oils were stored in their original light plastic bottles at 23 ± 2°C. All the solvents were of analytical grade or better and were obtained from Sigma‐Aldrich (St. Louis, MO) and Fisher Scientific (Fair Lawn, NJ).

### 2.2 Methods

#### 2.2.1 Oil extraction and margarine preparation

Oils: Njangsa seed oil (NSO) and bush mango kernel oil (BMO) were extracted as described (Arrey et al., 2022) using hexane and stored at −20°C until further analysis. The oils were used as‐is without refining. Formulated margarines: BMO and NSO (BN) or with soybean oil (SBO) (BS), coconut oil (CCO) and NSO (CN) or with soybean oil (CS) were prepared as described (Nadeem et al., 2017) using 82% oil phase, 1% lecithin, 0.6% salt, and 16.4% water. The oil phase included the hard stock: CCO or BMO, and the liquid fraction: SBO or NSO. The fully melted lipids and other ingredients were weighed into a 600‐mL beaker and homogenized using a hand‐held blender at medium speed for 4 min. The beaker containing the mixture was transferred to an ice bath and blender speed was reduced to the lowest and beaten until well combined. When the margarine solidified, it was transferred into a mold for shaping, then wrapped with parchment paper and stored at 4°C until needed. In addition to these formulated margarines, two commercial margarines CM1 and CM2 were used for comparison.

#### 2.2.2 Heat treatment and sampling

Aliquots of each oil and margarine were reserved as a control sample (without heat treatment). Sixty grams each of NSO, BMO, CCO, and SBO and 30 g of margarines: BN, BS, CN, CS, CM1, and CM2 were separately weighed and treated by heating in beakers at 130, 170, or 210°C (maintained at ±2°C). During heating, the samples were stirred periodically, and heating was monitored using an IR temperature thermometer (General No. IRT207 Secaucus, NJ). Aliquots of the oils were drawn at 10, 15, 20, and 120 min, and 10, 15, and 20 min for the margarine into amber vials. The vials were flushed with nitrogen and stored at −20°C until needed for analysis.

#### 2.2.3 Fatty acid composition

Fatty acid composition was analyzed using a modified AOCS Official Method Ce 2b‐11. In brief, 50 mg aliquots of the oils and margarines were weighed into screw‐cap glass vials and converted to their methyl ester derivatives using methanol containing 0.5 N sodium methoxide, and 2 mL hexane was added sequentially. An aliquot (25 μL) of the internal standard, heptadecanoic acid (C17) was then added. The vial was vortexed and then placed in an aluminum bead hot bath at 100°C for 10 min. After cooling, 250 μL of water was added, and the tubes were recapped, vortexed, and centrifuged for 3 min at 3000 rpm to separate layers. Aliquots of 50 μL hexane (upper) layer were transferred to glass vials. Analysis was carried out using a GC 2010 gas chromatograph (Shimadzu Corp., Columbia, MD), a ZB‐FAME 30 m fused silica capillary column (0.25 mm internal diameter and 0.2 μm film thickness; Phenomenex, Torrance, CA) coupled to a flame ionization detector. Run conditions were as follows: carrier gas, hydrogen; linear velocity, 22.0 cm/s; injector temperature, 260°C; oven program, the initial temperature of 100°C hold for 2 min, ramp temperature at a rate of 10°C/min to 140°C, then ramp at a rate of 3°C/min to 190°C, and finally, increased to 260°C at a rate of 30°C/min and hold for 2 min (total run time 30 min); and detector temperature, 240°C. A standard mixture of fatty acids (GLC 37, Supelco standards) was used to determine individual fatty acid response factors in CCO and BMO. The test samples were run in tandem with another standard mixture of fatty acids (15A standards, Nu‐Chek, Elysian, MN). Fatty acid composition was expressed as a percent of the total identified fatty acids.

#### 2.2.4 Oil quality parameters

The free fatty acid (FFA) content of the unheated (UH) oils, heat‐treated (HT) oils (120 min), and margarines (20 min) was determined per the modified AOCS Official Method Ca 5a‐40 for small sample sizes and expressed as oleic acid equivalent (Rukunudin et al. 1998).Peroxide value (PV) was determined per AOCS Offical Method Cd 8‐53 for reduced sample weight (0.25 g). The PV was expressed as mEq/kg (1 mmol/kg = 2 mEq O_2_/kg). Anisidine value (AnV) was determined using AOCS Offical Method Cd 18‐90. The total amount of intermediate polar compounds (peroxides and aldehydes) that result from lipid oxidation was used to calculate the total oxidation value (TOTOX) as: TOTOX = 2PV + AnV.

#### 2.2.5 Phytosterol content

The content of phytosterols was determined as described (Hwang et al., 2003). In brief, 100 mg aliquots of the oils and margarines were saponified using 33% (w/v) potassium hydroxide in ethanol at the ratio of 1:15.67 (v/v) with cholesterol as the internal standard. Then, 2 mL of potassium chloride and hexane were added and the mixture vortexed. After phase separation, the hexane layer was passed through an anhydrous sodium sulfate column to remove residual moisture. Chromatographic analysis was carried out using a GC 2010 chromatograph equipped with an auto‐injector and FID detector (Shimadzu Corp., Columbia, MD), AOC‐20i ZB‐5MS (5%‐phenyl‐arylene‐95%‐dimethylpolysiloxane)‐fused silica capillary column (30 m × 0.25 mm id × 0.25 μm film thickness; Phenomenex, Torrance, CA). The oven temperature was initially 278°C and increased to 286°C at a rate of 2.5°C/min. After holding for 6 min, the temperature was increased to 278°C at 40°C/min and held for 8 min. The injector and detector temperature were maintained at 295°C and split ratio was 1:50. The carrier gas was helium at a flow rate of 30 mL/min. The phytosterols were identified by comparing their retention times with standards (Sigma‐Aldrich, St. Louis, MO).

#### 2.2.6 Statistical analysis

All analyses were done in triplicate (unless otherwise stated) and results were expressed as mean ± standard deviation. One‐way analysis of variance (ANOVA) and their statistical significance (*p* ≤ .05) were applied using Tukey's post hoc analysis (SPSS version 25). Alpha value was set at (p ≤ 0.05).

Principal component analysis (PCA), partial least‐squares discriminant analysis (PLS‐DA), and hierarchical clustering were calculated and visualized in R 4.3.0 (The R Foundation for Statistical Computing, Vienna, Austria). PLS‐DA was done using R package “mixOmics” 6.22.0. Spearman's rank correlation coefficients (*ρ*) and their significance were calculated with R package “correlation” 0.8.4 and then visualized with R package “corrplot” 0.92. Centering and scaling were applied for PCA, PLS‐DA, and correlation data, however, for PLS‐DA and correlation, it was applied to each sample type independently. Complete linkage method was used for clustering.

## 3 RESULTS AND DISCUSSION

**FIGURE 1:**
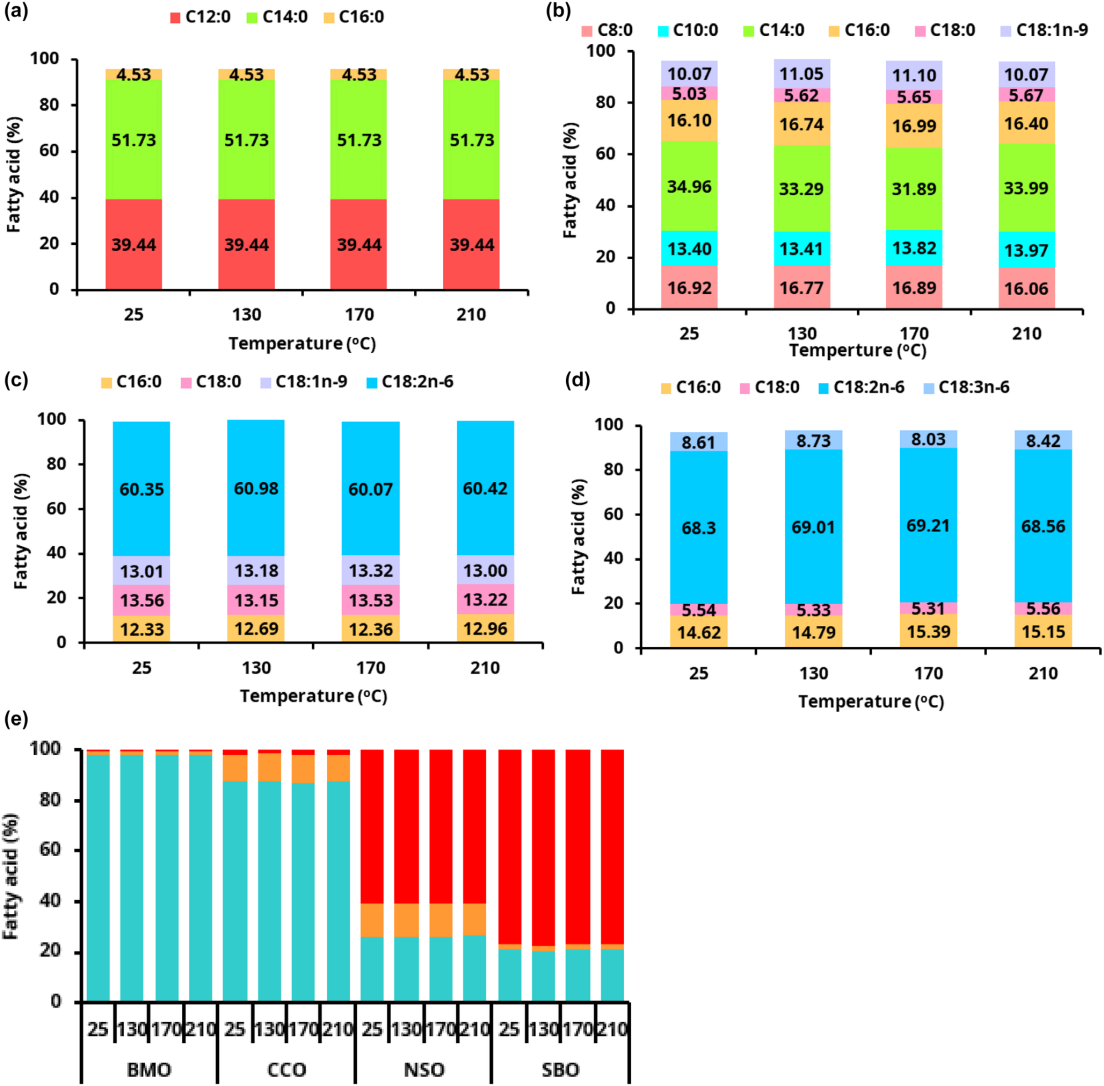
Changes in fatty acid composition of (a) bush mango oil (BMO), (b) coconut oil (CCO), (c) njangsa seed oil (NSO) and (d) soybean oil (SBO) and (e) fatty acid classes without or after 120 min of heat treatment. Caprylic acid (C8:0), capric acid (C10:0), lauric acid (C12:0), myristic acid (C14:0), palmitic acid (C16:0), stearic acid (C18:0), oleic acid (C18:1n‐9), linoleic acid (C18:2n‐6), and γ‐linolenic acid (C18:3n‐6). Caproic acid (C6:0), tridecylic acid (C13:0), and arachidic acid (C20:0) (<3%) are not shown.

**FIGURE 2:**
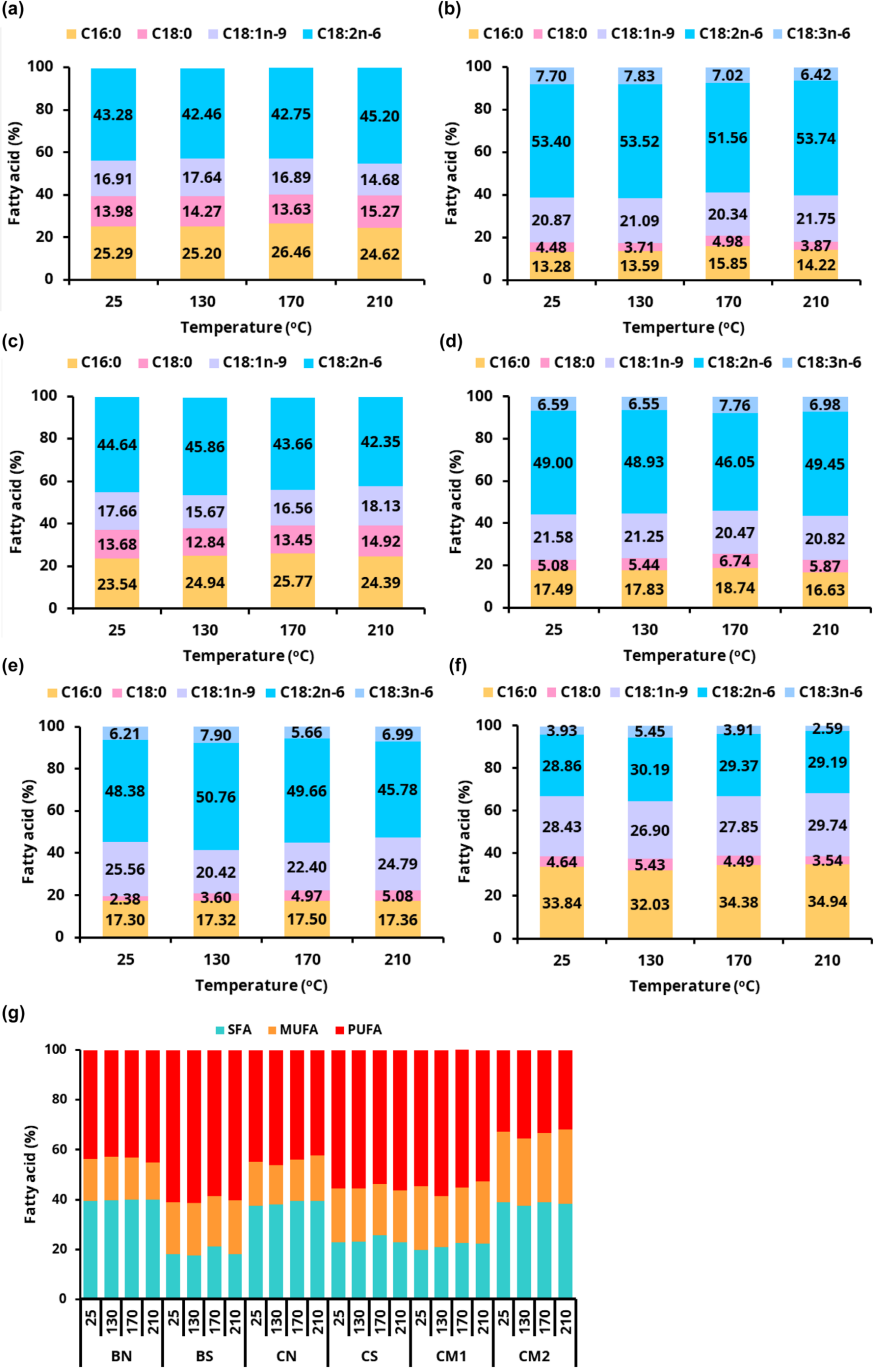
Changes in fatty acid composition of margarines formulated from blends of (a) bush mango oil (BMO) and njangsa seed oil (NSO) (BN), (b) BMO and soybean oil (SBO) (BS), (c) coconut oil (CCO) and NSO (CN), (d) CCO and SBO (CS) and commercial margarines (e) CM1 and (f) CM2 and (g) fatty acid classes (SFA, MUFA and PUFA) without or after 20 min of heat treatment. Palmitic acid (C16:0), stearic acid (C18:0), oleic acid (C18:1n‐9), linoleic acid (C18:2n‐6), and γ‐linolenic acid (C18:3n‐6). Arachidonic acid (C20:0) (<1%) is not shown.

**FIGURE 3:**
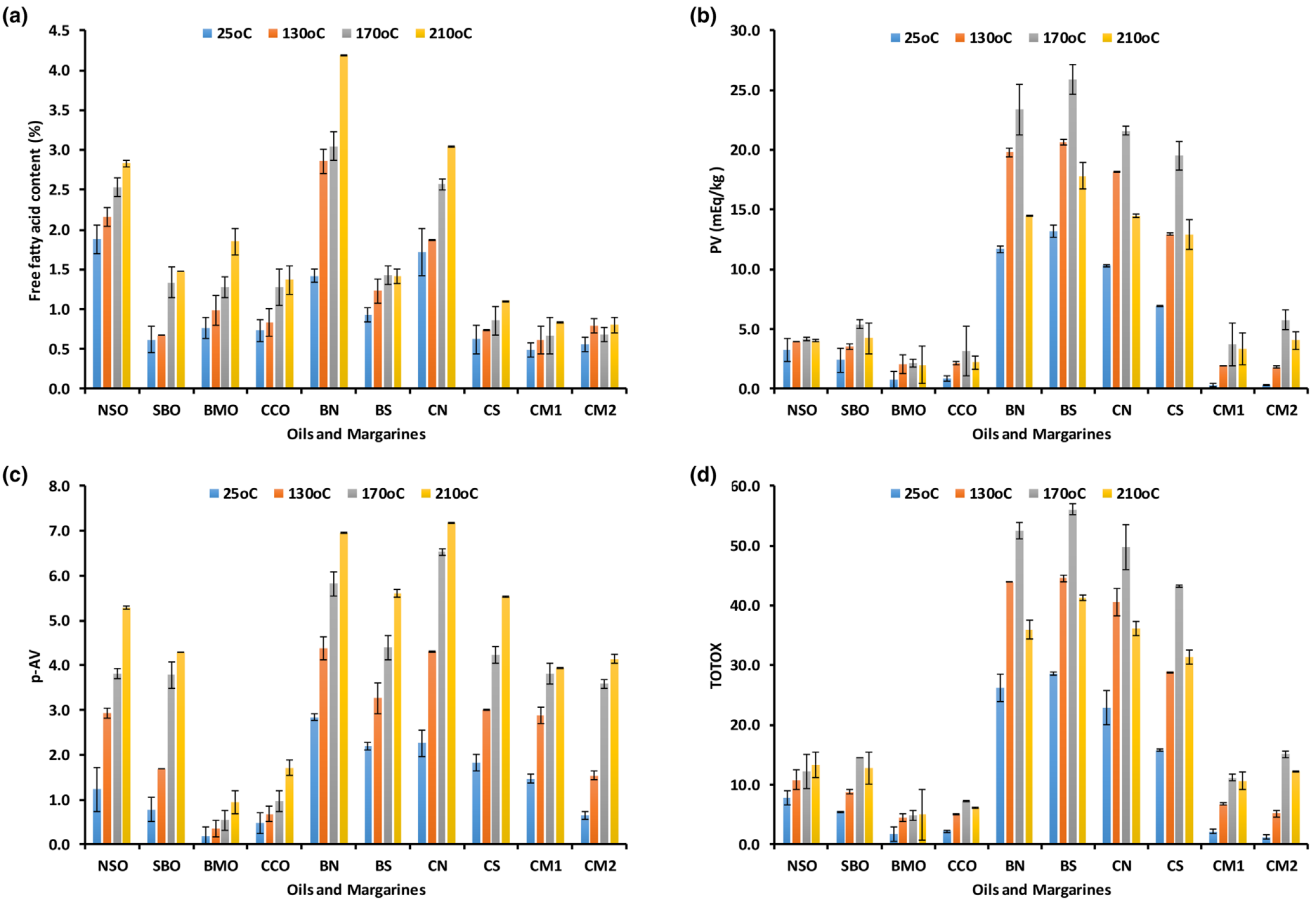
Changes in free fatty acid content (%) (a), peroxide value (mEq/kg) (b), para‐anisidine value (c), and TOTOX (d) of unheated (25°C) oils: Njangsa seed (NSO), bush mango kernel (BMO), soybean (SBO) and coconut (CCO), and heated (130, 170, and 210°C) for 120 min, and margarines formulated from blends of BMO and NSO (BN), BMO and SBO (BS), CCO and NSO (CN), CCO and SBO (CS), and commercial margarines (CM1 and CM2) heated at the same temperatures for 20 min.

**FIGURE 4:**
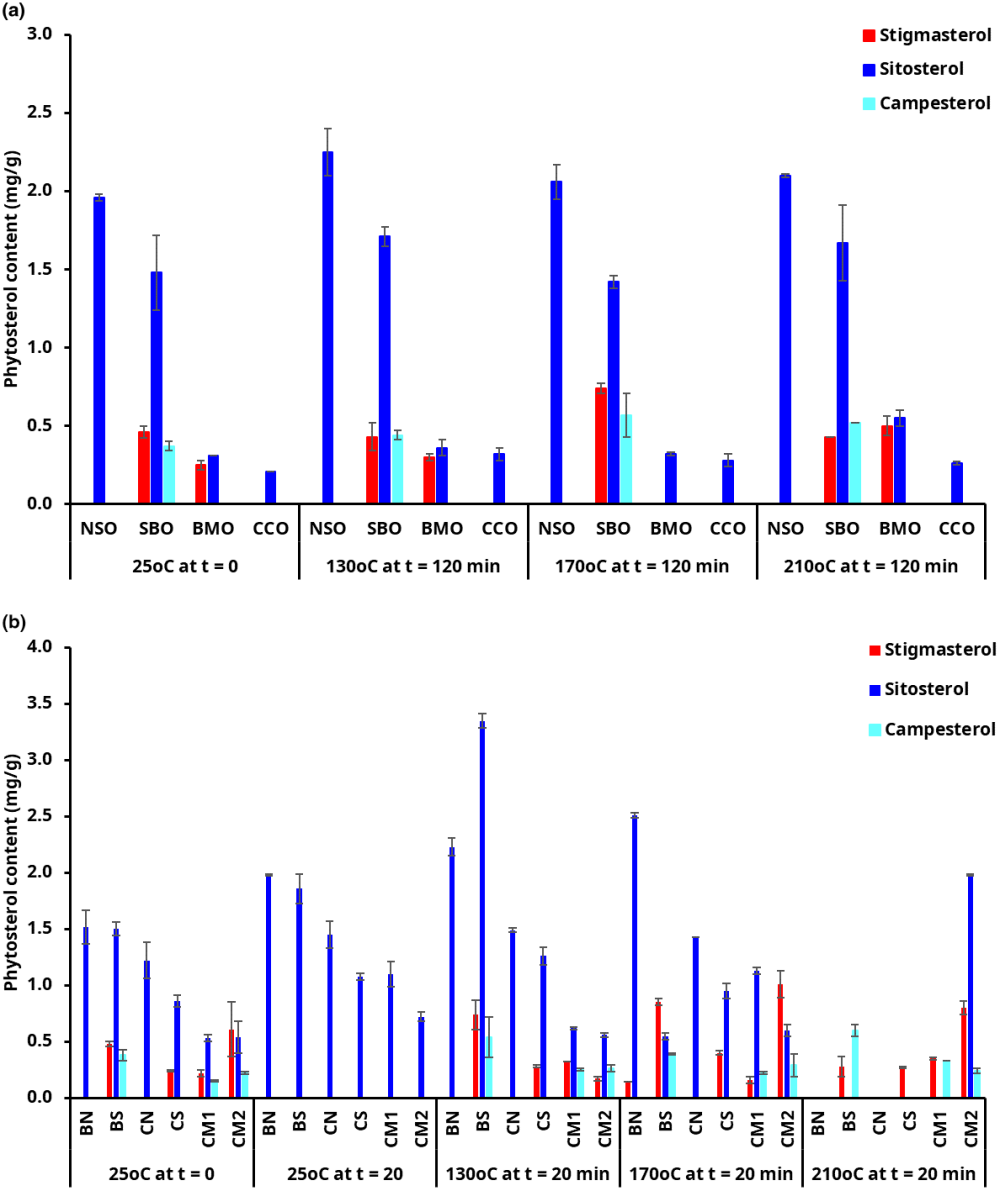
Changes in phytosterol content in (a) bush mango oil (BMO), coconut oil (CCO), njangsa seed oil (NSO) and soybean oil (SBO) without or after 120 min of heat treatment, and (b) margarines formulated from blends of BMO and NSO (BN), BMO and SBO (BS), CCO and NSO (CN), CCO and SBO (CS), and commercial margarines (CM1 and CM2) without or after 20 min of heat treatment.

The changes in quality indices and the composition of fatty acid and phytosterol content of the control/unheated (UH) and heat‐treated (HT) oils and margarines are presented in Figures 1, 2, 3, 4.

### 3.1 Changes in fatty acid composition

The profile of fatty acids is an important determinant of the quality and stability of oils. The changes in fatty acid composition of the four oils during UH and HT are shown in Figure 1a–e. SFAs such as lauric (C12:0) and myristic (C14:0) were the main fatty acids in BMO (~97%) (Figure 1a), and caprylic (C8:0), myristic (C14:0), and palmitic (C16:0) in CCO (~87%) (Figure 1b). The fatty acid profiles of BMO (Figure 1a) and CCO (Figure 1b) were consistent with earlier studies (Ogunsina et al., 2012). The high content of SFAs makes these two oils quite stable during heat treatment (Figure 1e). In contrast, PUFAs were the main fatty acids in NSO (~61%) (Figures 1c and e) and SBO (~77%) (Figures 1d and e). Apart from stearic acid (C18:0) in NSO and palmitic acid (C16:0) in SBO, linoleic acid (C18:2n‐6) was the main PUFA in both NSO and SBO, making them more susceptible to oxidation. Among all other factors, the degree of unsaturation is an important determinant of oxidative stability (Kozłowska et al., 2019; Sharoba & Ramadan, 2017; Silva et al., 2022). The content of myristic acid (C14:0), the main SFA in BMO did not change significantly during the three different HTs at 130, 170, and 210°C (Figure 1a). Similarly, significant differences were not found in the content of lauric acid (C12:0), the second main SFA in BMO during the three HTs. This trend was also observed in all the other fatty acids of all the oils and margarines, where the content increased, decreased, or fluctuated slightly with HT but was not statistically significant. A number of studies reported a reduction in PUFAs and a corresponding increase in SFAs with increasing frying time (Hicham et al., 2014; Schubert et al., 2022; Sharoba & Ramadan, 2017; Silva et al., 2022; Wongjaikham et al., 2022). Ben Hammouda et al. (2018) demonstrated that blends of MUFA‐rich refined olive pomace oil with SFA‐rich oils like refined palm oil showed better performance during repeated frying cycles. Zribi et al. (2016) determined that the blending of MUFA‐rich refined olive oil with SFA oils like refined palm oil (80:20) resulted in the highest chemical stability during the frying process, while a similar blending ratio of refined soybean oil with refined palm oil presented the lowest stability.

The relatively shorter HT time used in this study compared to several hours of treatment reported in the above‐mentioned studies may account for the contrast. Figure 2a–f show the changes in the fatty acid composition and fatty acid classes (Figure 2g) of the margarines during the various heat treatments. The content of the various fatty acids did not change significantly with heat treatment. The relatively higher content of stearic acid (C18:0) and lower amounts of PUFAs, such as linoleic (C18:2) and linolenic (C18:3) in BN (Figure 2a) and CN (Figure 2c) margarines, may account for the relatively higher stability of these margarines during HT. A few minor fatty acids, such as C20:0 in BN, BS, CM1, and CM2 margarines, were undetectable below the limit at high temperatures. The content of fatty acids of both NSO and SBO in this study were higher than previously reported (Ezekwe et al., 2014). This may be due to the different analytical method, standards and columns used.

### 3.2 Changes in quality indices

The FFA content of the control/UH and HT oils and margarines were in the range of 0.62–2.83 and 0.49%–4.19%, respectively (Figure 3a). The change in FFA content was considerably higher in PUFA‐rich oils (NSO and SBO) than observed in the more saturated oils (BMO and CCO) with increasing HT (Figure 3a). The higher FFA in SBO is characterized by a high amount of PUFA (~77%), with the largest proportion contributed by linoleic acid (~68%), which may have had a higher influence on oxidation than antioxidants such as α‐tocopherol present in SBO (and NSO) as well as the initial oxidative state of the oil. Conversely, the content of FFA in the different margarines increased during HT. Prior to HT, FFA content was lowest in CS margarine (0.62%), followed by BS, BN, and CN margarines with FFA content of 0.93%, 1.42%, and 1.72%, respectively. BN margarine had the highest content of FFA after HT at 210°C as well as the highest increase in FFA content during HT. The FFA content in BN margarine changed by 195% between the control and HT at 210°C, while BS, CN, and CS margarines changed by 51%, 77%, and 79%, respectively. The difference may be attributable to the different oils used to formulate the margarines. Similarly, the high PUFA content of CN margarine may explain its high FFA content as well as blending‐induced oxidation, pre‐existing oxidation, and amount of impurities present (Silva et al., 2022; Wongjaikham et al., 2022). Although the FFA values seen in this study are higher than those reported in some previous studies, the percent change from control to 210°C (9%–195%) was lower than in previous studies (88%–457%) (Herchi et al., 2016; Hicham et al., 2014; Latha & Nasirullah, 2014).

PV is used as an index of hydroperoxides formed as primary oxidation products. The PV assayed in all the oils and three of the margarines were below the CODEX limit of 10 meq O_2_/kg oil (Figure 3b). The PV generally increased slightly in the oils and margarines at lower HT (130°C), with the increase becoming more intense at higher temperature (170°C) (Figure 3b). Differences between PV in each of the oils and margarines were statistically significant. The lowest PV in the UH oils and formulated margarines was determined in BMO and CS margarines as 0.80 and 6.96 meq O_2_/kg oil, respectively. The high amounts of SFAs in BMO and CCO may explain their low PV in their control/UH samples. NSO characterized by high PUFAs had the highest PV (3.28 meq O_2_/kg oil) among the oils after formulation (without HT) and increased to 4.20 meq O_2_/kg oil after HT at 170°C. As previously reported, both the rate of formation and amount of primary oxidation compounds accumulated at the end of the treatment period increase with increasing degree of unsaturation (Kozłowska et al., 2019). PV increased with increasing HT in all the other oils and margarines up to 170°C (Figure 3b). At 210°C, lower amount of PV was recorded. This could be explained by the decomposition of the hydroperoxides at temperatures above 170°C and the insensitivity of the PV test to measure the decomposed products. PV changed by 96% and 180% between control and HT at 170°C in BS and CS margarines, respectively, and the high SFA content may explain this trend. PV changed by 100% and 109% between the control and HT at 170°C in BN and CN margarines, respectively. Compared to the formulated margarines, the PV of the commercial margarines were relatively low in both the UH and HT samples.

It is generally not possible to predict the best indicator of lipid oxidation, and any attempt to characterize this process will likely require multiple tests. The pattern of change in the oils and margarines was also followed by measuring changes in the accumulation of secondary oxidation products, mainly 2‐alkenals and 2,4‐alka‐dienals. The AnV is routinely used as a measure of aldehydes formed as secondary oxidation products from the decomposition of hydroperoxides. The low aldehyde concentrations observed in this study may be the result of limited oxidation or the aldehydes may have volatilized. Lower AnV was assayed in the UH oils (0.17–0.78) (Figure 3c). The AnV of the oils tended to moderately increase with HT and increased up to 0.95 and 5.29 in BMO and NSO, respectively, at 210°C (Figure 3c). The AnV of NSO and SBO was only ~5–7 and ~6–8 fold higher than BMO, respectively. Previous studies have also reported that while oils and margarines with a high degree of unsaturation are more susceptible to oxidation at storage temperature, they tend to have better resistance to thermal oxidation because the peroxides formed are quickly destroyed as temperature increases, thereby boosting their resistance to becoming rancid (Kasbo, 2011). Significant differences were found in the AnV of the margarines with increasing HT in agreement with Latha and Nasirullah (2014), who reported increasing AnV with increasing heating time. Prior to HT, both BN and CN margarines had significantly higher AnV than the other margarines and this may be due to the high proportion of linoleic acid (C18:2) in NSO. With the exception of CM1 and CM2, the AnV in the other margarines was higher than previously reported in margarine formulated with palm oil and sunflower oil (AnV = 1.50) (Azizkhani & Zandi, 2010). The high stability of CM1 and CM2 may be due to the higher content of SFAs and the inclusion of antioxidants in commercial margarines.

Prior to HT, the oils with the lowest (1.77) and highest (7.80) TOTOX were BMO and NSO, respectively (Figure 3d). A TOTOX of <10 is characteristic of fresh, high‐quality oils. Generally, the lower the TOTOX, the better its stability (Xu et al., 2015). This agrees with the results of the fatty acid composition, suggesting higher oxidative stability of BMO compared to the more unsaturated NSO. In another study, authors ascribed the lower TOTOX in camellia oil compared to palm and peanut oils to its high MUFA content (Xu et al., 2015). The TOTOX of NSO and BMO increased by nearly two‐ to threefold between the control and HT at 210°C. The oils and margarines ranked in order of increasing TOTOX at the highest heat treatment are as follows: BMO < CCO < CM1 < CM2 < SBO < NSO < CS < BN < CN < BS.

### 3.3 Changes in phytosterol content

The changes in the content of phytosterols, including β‐sitosterol, stigmasterol, and campesterol, are presented in Figure 4. Phytosterols are plant‐derived cholesterol analogs whose application has received some attention due to their perceived benefits. Previous studies have reported the effect of phytosterol supplementation on plasma LDL cholesterol concentrations with cholesterol‐lowering effects of even moderate phytosterol intake in humans—such as that obtained from a diet rich in plant‐based foods (Poli et al., 2021; Racette et al., 2010). There are limited studies of changes in the content of phytosterols in the oils and their blend used in this study for adequate comparison. The total phytosterol content in the unheated CCO, BMO, NSO, and SBO were 0.21, 0.56, 1.96, and 2.32 mg/g, respectively, and 0.90 (CM1), 1.10 (CS), 1.22 (CN), 1.37 (CM2), 1.75 (BN), and 2.72 (BS) in the margarines (Figure 4b). The total phytosterol content in both SBO and CCO in this study was ~4‐fold lower than reported by Sabir et al. (2003). All three phytosterols were observed in SBO and margarine blend and the content in the margarine was comparable with the two commercial margarines (CM1 and CM2).

β‐sitosterol was the most abundant phytosterol in all the analyzed samples, followed by stigmasterol and campesterol (Figure 4) similar to the study by Kmiecik et al. (2020). Pre‐HT: 0.21, 0.31, 1.48, and 1.96 mg/g of sitosterol were determined in CCO, BMO, SBO, and NSO, respectively. The amount of β‐sitosterol in NSO in this study was higher than the 527.23 μg/g previously reported (Yeboah et al., 2011). This might be explained by the differences in the extraction process, storage, and analytical methods used. No detectable levels of stigmasterol were found in NSO, CCO, and their blend (CN margarine). Campesterol was not detected in BMO, contrary to a study where all three sterols, sitosterol (46.7%–76.0%), stigmasterol (11.5%–22.7%), and campesterol (6.80%–11.50%), were observed (Gaydou & Bouchet, 1984). In addition, campesterol was not detected in NSO, CCO, and blends containing these two oils as well as BMO (CN, CS, and BN margarines). Stigmasterol was determined in only BMO, SBO, and their blends (BN, BS, and CS margarines). CM1 contained about threefold higher amount of stigmasterol than CM2 and nearly equal amounts of campesterol and sitosterol.

In general, there was a slight increase in sterol content in all the oils with HT (Figure 4a). The content of β‐sitosterol increased by nearly 16% and 29% in SBO and NSO, respectively. Similar trends were observed in some of the oils and margarines with HT, while others fluctuated. This was unexpected since sterols are broken down into sterol oxidation products under HT (Chew et al., 2018; da Silva et al., 2022a, 2022b). Sterol oxidation is a free radical chain reaction where the hydrogen atoms are transferred from the attack sites (C3, C7, and C25) in the sterol molecule. The oxidized sterols (oxysterols) formed are similar to sterol, with an additional group such as hydroxy, keto, and epoxy (Lengyel et al., 2012; Schroepfer & Wilson, 2000; Silva et al., 2021; Johnsson and Dutta, 2005). The unseparated oxysterol and unoxidized sterols could have coeluted and overlapped since our column (30 m) was shorter than in the previous studies (25–60 m) (Johnsson and Dutta, 2005; Ubhayasekera, 2009) and (100 m) (Kmiecik et al., 2020). In addition, our column, a SHRXI‐5MS phase nonpolar capillary column with methyl groups attached, may have resulted in the coelution of some compounds (dos Santos et al., 2014). The higher but nonsignificant changes observed in the content of phytosterols in the margarines with HT may also be due to increased oxidation at the interface compared to the oil phase alone (Bai et al., 2021). The composition of the oil may also have influenced the stability of the phytosterols. In a previous study, the composition of the oil was found to be unrelated to the stability of the phytosterols (Winkler et al., 2007) and not the only factor that affects the oxidation of phytosterols in foods during heating (Kmiecik et al., 2020). Moreover, there were no significant differences in sterol content within the same treatment temperatures and study duration. This may be due to the much shorter HT time used in this study compared to the several hours of treatment reported in the above‐mentioned studies. It is also possible that the phytosterols were not consumed rapidly during HT and the higher stability of the phytosterols compared to the unsaturated fatty acids or other lipids in the samples. The changes in phytosterol content may have also been influenced by the conditions of the heating process such as the use of beakers in this study as opposed to metal pans in other studies, volume of sample heated, and the type of heating method (deep‐frying vs. pan‐frying) (Kmiecik et al., 2020).

### 3.4 Correlation, PCA, PLS‐DA, and clustering analyses

**FIGURE 5:**
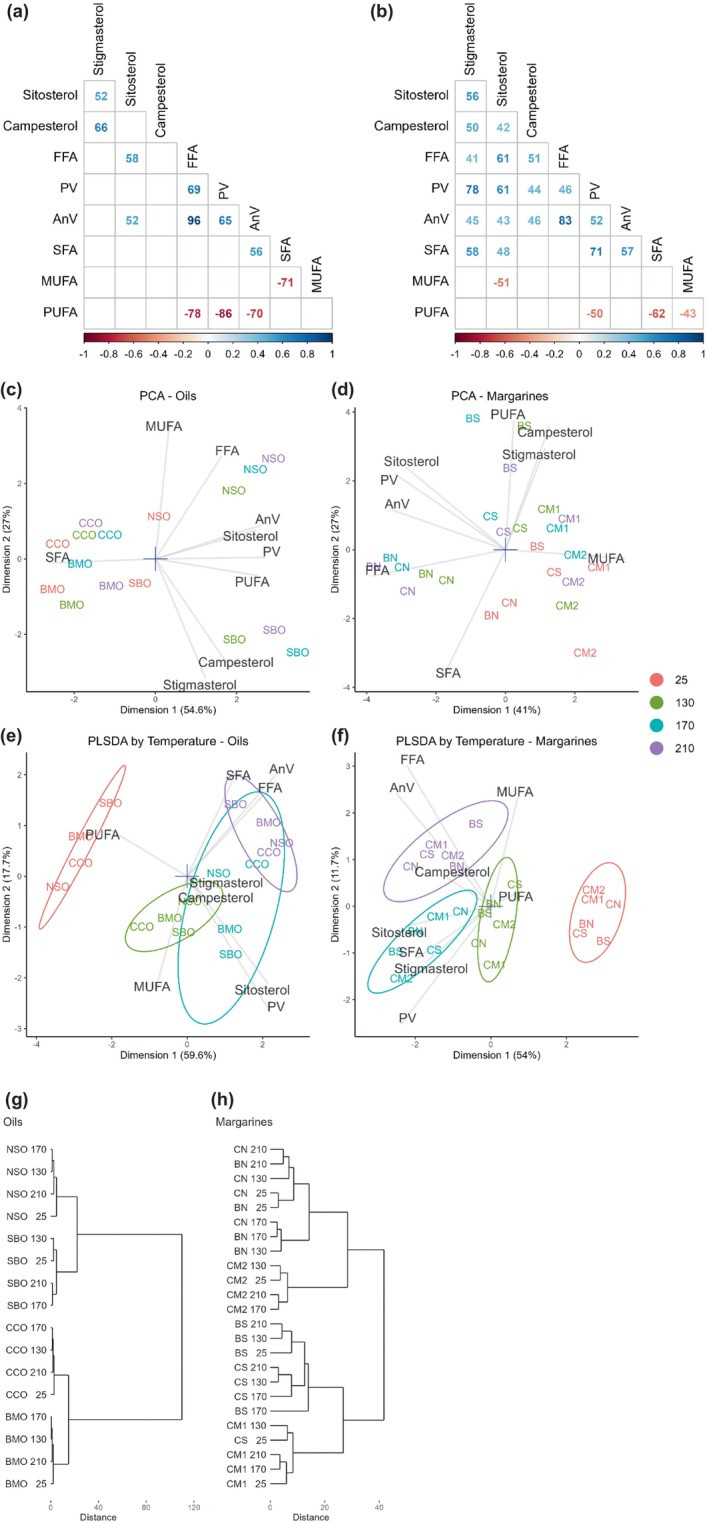
Statistically significant Spearman’s correlations for oils (a) and margarines (b), including before and after heat treatment (multiplied by 100 for brevity). Data was centered and scaled for each sample separately. Principal component analysis (PCA) (c and d) and partial least‐squares discriminant analysis (PLS‐DA) (e and f) biplots for the four oils and six margarines without (25oC) or after heat treatment (130, 170, and 210oC) for 120/20 min. For PLS‐DA, data was centered and scaled for each sample separately. Ellipses indicate 95% confidence regions for cluster centers. Hierarchical clustering dendrogram representing similarities in oils (g) and margarines (h) without (25oC) or after heat treatment (130, 170, and 210oC) for 120/20 min. Quality parameters: free fatty acid (FFA), peroxide value (PV), para‐anisidine value (AnV); and fatty acid classes: polyunsaturated fatty acids (PUFA), monounsaturated fatty acids (MUFA), and saturated fatty acids (SFA). Oils: Njangsa seed (NSO), bush mango kernel (BMO), soybean (SBO) and coconut (CCO, and margarines formulated from blends of BMO and NSO (BN), BMO and SBO (BS), CCO and NSO (CN), CCO and SBO (CS), and commercial margarines (CM1 and CM2).

The correlations among the quality parameters, fatty acid classes, and phytosterols content are shown in Figure 5a for the oils and in Figure 5b for the margarines. For the calculation, the data were first centered and scaled for each sample separately, so it could be interpreted as the average trend among all the samples, including changes caused by heat treatment. In the oils, an increasing SFA content negatively correlated with MUFA, while in the margarines, SFA content was mostly negatively correlated with PUFA. In the oils, the quality parameters were strongly negatively correlated with PUFA, but not with MUFA. In contrast, these correlations were much weaker or absent in the margarines, suggesting higher thermal stability. The highest correlation coefficient was found between AnV and FFA in both the oils and margarines. Phytosterols had no strong correlations with the fatty acid classes in the oils, but in margarines were moderately positively correlated with the quality parameters with heat treatment.

PCA was able to describe the nine parameters using two principal components with explained variance of 81.6% and 68% for the oils (Figure 5c) and margarines (Figure 5d), respectively. Some clustering according to the sample type and treatment temperature was obtained. NSO and SBO heat‐treated at 130, 170, and 270°C were the most distinct from the rest of the oils; the heat‐treated margarines BS, CN, and BN showed similar behavior. According to the PCA, NSO and SBO had the largest shift in composition with heat treatment, indicating their lower stability in comparison to BMO and CCO. This can be explained by the much higher concentration of unsaturated fatty acids in NSO and SBO. Since the margarines were prepared by blending the oils, their overall composition was less diverse, thus the shifts observed on the biplot due to heat treatment were of a similar scale for all the samples.

To identify the effect of heat treatment more clearly, we performed PLS‐DA. The data were centered and scaled for each sample separately to eliminate the differences due to the sample composition and show only the effect of temperature. As can be seen in Figure 5e,f, the samples formed well‐defined groups by temperature. The classification error rates of PLS‐DA models based on the Mahalanobis distance obtained by fourfold cross‐validation repeated 10 times were 0.21 ± 0.04 and 0.16 ± 0.07, for oils and margarines, respectively. The untreated samples were the most distinct, especially the untreated oils that had a relatively higher PUFA content, but the absolute difference was small. Heat treatment of oils and margarines, in general, caused an increase in phytosterols, PV, FFA, and AnV; however, the effect depended on the temperature. Oils treated at 210°C, in comparison to 130°C, showed lower MUFA and higher SFA, FFA, and AnV. However, in margarines, the same increase in temperature caused an increase in FFA and AnV only. Margarines heated at 170°C, in comparison to the other temperatures, showed generally lower MUFA and higher PV, SFA, stigmasterol, and sitosterol content. This trend was also seen in the oils, but not as prominently. This analysis suggests that the composition of oils and margarines and the treatment temperature influence the changes caused by heat‐induced oxidation.

A hierarchical clustering dendrogram was constructed to show the similarities among the samples (Figure 5g,h). Corroborating the results of PCA, the distances between the margarine samples were smaller and their clusters were not well defined. Oils, on the other hand, were clearly differentiated by their blends regardless of the treatment temperature, although untreated samples were the most different in each cluster (Figure 5a). Higher‐level clusters of oils formed according to their fatty acid composition, putting NSO together with SBO and CCO with BMO. In margarines, higher‐level clusters grouped margarines CN and BN, both formulated with NSO, together with the commercial margarine CM2, while CS and BS, containing SBO, were more akin to CM1. Within those clusters, commercial margarine samples were still clearly distinct, but the blends were separated weakly.

Considering the overall results of correlation, PCA, PLS‐DA, and clustering analyses, the pattern of oxidative changes in the different oils and margarines strongly depends on their composition. Almost all measured parameters showed non‐linear trends as specific components were either additionally produced or degraded depending on the temperature. Only AnV increased with increasing in treatment temperature. Even though AnV was strongly positively correlated with FFA, suggesting that they both can be used to monitor the stability during heat treatment, for margarines BS and CM2, the changes in FFA content were inconsistent. According to the clustering analysis, both commercial margarines had the smallest overall changes after heat treatment, yet fatty acid composition of CM1 was not different from CS and BS, which had lower stability, thus other factors may also be important, like the presence of antioxidants in the commercial margarine, heating temperature, time, and type of phytosterols. High scores of the quality parameters were determined in the PUFA‐rich oils and blends which also showed higher scores.

## 4 CONCLUSIONS

The oils and margarines exhibited various oxidative stabilities due to their different fatty acid composition, treatment temperature, and duration of treatment. Oils with a higher proportion of SFAs exhibited higher oxidative stability. The most appropriate heating temperature for the oils and margarines were 130 and 170°C up to 20 and 120 min, respectively. Phytosterol composition and content also vary among the oils and margarines. The high phytosterol content determined with heat treatment suggests that further studies are necessary to concurrently determine phytosterol oxidation products. The correlation and clustering analyses suggest that the pattern of oxidative changes in the different oils and margarines strongly depends on their composition. This study provides valuable information on the behavior of these oils and their blends, during heat treatment, and potential losses in food quality to guide their utilization in product development.

## AUTHOR CONTRIBUTIONS

**Anh T. L. Nguyen:** Formal analysis (equal); investigation (lead); methodology (equal); writing – original draft (equal); **Aleksei Kaleda:** Data curation (supporting); visualization (supporting); **John O. Onuh:** Writing – review and editing (supporting); **Alberta N. A. Aryee:** Conceptualization (lead); formal analysis (equal); funding acquisition (lead); project administration (lead); resources (lead); supervision (lead); validation (lead); writing – original draft (equal); writing – review and editing (lead).

## CONFLICT OF INTEREST STATEMENT

The authors declare no conflict of interest.

## Data Availability

Data are available within the article or its supplementary materials.
